# A bloom of a single bacterium shapes the microbiome during outdoor diatom cultivation collapse

**DOI:** 10.1128/msystems.00375-25

**Published:** 2025-05-14

**Authors:** Naomi E. Gilbert, Jeffrey A. Kimbrel, Ty J. Samo, Anthony J. Siccardi, Rhona K. Stuart, Xavier Mayali

**Affiliations:** 1 Physical and Life Sciences Directorate, Lawrence Livermore National Laboratory 4578, Livermore, California, USA; 2 Department of Biology, Georgia Southern University 7604, Statesboro, Georgia, USA; University of Minnesota Twin Cities, St. Paul, Minnesota, USA

**Keywords:** *Kordia*, biofuel ponds, metagenomics, metaproteomics, cross-feeding

## Abstract

Algae-dominated ecosystems are fundamentally influenced by their microbiome. We lack information on the identity and function of bacteria that specialize in consuming algal-derived dissolved organic matter in high algal density ecosystems such as outdoor algal ponds used for biofuel production. Here, we describe the metagenomic and metaproteomic signatures of a single bacterial strain that bloomed during a population-wide crash of the diatom, *Phaeodactylum tricornutum,* grown in outdoor ponds. 16S rRNA gene data indicated that a single *Kordia* sp. strain (family *Flavobacteriaceae*) contributed up to 93% of the bacterial community during *P. tricornutum* demise. *Kordia* sp. expressed proteins linked to microbial antagonism and biopolymer breakdown, which likely contributed to its dominance over other microbial taxa during diatom demise. Analysis of accompanying downstream microbiota (primarily of the *Rhodobacteraceae* family) provided evidence that cross-feeding may be a pathway supporting microbial diversity during diatom demise. *In situ* and laboratory data with a different strain suggested that *Kordia* was a primary degrader of biopolymers during algal demise, and co-occurring *Rhodobacteraceae* exploited degradation molecules for carbon. An analysis of 30 *Rhodobacteraceae* metagenome assembled genomes suggested that algal pond *Rhodobacteraceae* commonly harbored pathways to use diverse carbon and energy sources*,* including carbon monoxide, which may have contributed to the prevalence of this taxonomic group within the ponds. These observations further constrain the roles of functionally distinct heterotrophic bacteria in algal microbiomes, demonstrating how a single dominant bacterium, specialized in processing senescing or dead algal biomass, shapes the microbial community of outdoor algal biofuel ponds.

**IMPORTANCE:**

Aquatic biogeochemical cycles are dictated by the activity of diverse microbes inhabiting the algal microbiome. Outdoor biofuel ponds provide a setting analogous to aquatic algal blooms, where monocultures of fast-growing algae reach high cellular densities. Information on the microbial ecology of this setting is lacking, and so we employed metagenomics and metaproteomics to understand the metabolic roles of bacteria present within four replicated outdoor ponds inoculated with the diatom *Phaeodactylum tricornutum*. Unexpectedly, after 29 days of cultivation, all four ponds crashed concurrently with a “bloom” of a single taxon assigned to the *Kordia* bacterial genus. We assessed how this dominant taxon influenced the chemical and microbial fate of the ponds following the crash, with the hypothesis that it was primarily responsible for processing senescent/dead algal biomass and providing the surrounding microbiota with carbon. Overall, these findings provide insight into the roles of microbes specialized in processing algal organic matter and enhance our understanding of biofuel pond microbial ecology.

## INTRODUCTION

Algal-bacterial interactions fundamentally influence aquatic biogeochemistry and ecology (1–4). Algae support a diverse bacterial community (the algal microbiome) by exuding dissolved organic matter (DOM), and bacteria support algae through metabolite production and nutrient remineralization (5–11). Bacteria can also negatively impact algal hosts through competition or directly via algicidal mechanisms (10, 12, 13). A better understanding of the feedback resulting from algal-bacterial interactions during algal growth and demise is needed to predict the role of the algal microbiome in natural and engineered ecosystems. Moreover, uncovering how bacteria interact with each other within the algal microbiome will allow more accurate quantification of resource recycling across the algal lifecycle.

Algal microbiomes are functionally diverse, leading to partitioning of DOM processing and assimilation (14–16). Furthermore, a temporal succession of different bacterial taxa is well documented within marine algal blooms (17–20). Generally, in early algal growth, the DOM pool is composed of low molecular weight molecules (e.g., amino/organic acids, sugars) exuded by the algae (2, 14, 21). These, in turn, enrich for small-molecule users such as the *Rhodobacteraceae*, a group commonly found in algal microbiomes (14, 22). During algal demise, high molecular weight molecules (e.g., polysaccharides, proteins, lipids) from lysis or exudation dominate the DOM pool (14, 23) and are directly consumed by biopolymer degraders like the *Flavobacteriaceae* (19, 22, 24). Changes in DOM composition over time lead to a predictable succession of bacteria along the algal lifecycle.

The mass cultivation of microalgae in outdoor raceway ponds is considered to be an economically viable option for the production of biofuels, a renewable source of energy (25, 26). However, these systems are susceptible to changes in the surrounding environment, increasing the potential for system-wide algal pond crashes (27). These crashes can result from abiotic (e.g., temperature fluctuations and other suboptimal weather conditions) and/or biotic (e.g., viral, bacterial, eukaryotic infection) factors, all of which can have differing downstream effects on algal pond biogeochemistry (27, 28). However, the agent(s) causing pond crashes are rarely identified despite the need to mitigate these events. Moreover, because pond biogeochemistry is majorly dependent on the activity of heterotrophic bacteria via remineralization of carbon and nitrogen, for example, an understanding of how the microbiome recycles residual resources following pond crashes is needed.

Here, we document the microbial underpinnings associated with an algal population crash that occurred during an outdoor cultivation effort. We followed a month-long time course of algal raceway ponds inoculated with two distinct host algae: *Phaeodactylum tricornutum* (a diatom) and *Microchloropsis salina* (a chlorophyte). Using molecular tools (16S rRNA gene amplicon sequencing, metagenomics, and proteomics), we characterized the microbiome dynamics across time for the two algal hosts. A crash was observed only in *P. tricornutum* ponds after ~29 days of cultivation, which corresponded with a bloom in a single amplicon sequence variant assigned to the genus *Kordia* (family *Flavobacteriaceae*). Although we were unable to isolate the bacterium to test whether it could cause algal demise in the laboratory, we suspected that this bacterium contributed to *P. tricornutum* demise, as strains within the *Kordia* genus are capable of killing this model diatom (29) and other algae (30). Nevertheless, the profound shift in the microbiome during the pond crash led us to investigate potential mechanisms contributing to *Kordia*’s dominance and its downstream impact on the surrounding microbiota. We initially hypothesized that microbial taxonomic and functional composition would mirror DOM availability along the algal life cycle. We also performed a laboratory experiment to help explain patterns observed in the community-wide response to algal demise, suggesting cross-feeding is a mechanism in structuring the microbial community when algae die, specifically between *Flavobacteriaceae* and *Rhodobacteraceae*. Collectively, these results provide insight into the ecophysiology of *Kordia* sp. and emphasize the importance of considering the role of bacterial-bacterial interactions within the algal microbiome, both in nature and industrial settings, to understand the fate of algal-derived carbon.

## MATERIALS AND METHODS

### Algal raceway pond time series design

Eight 557 L algal raceway (ARW) ponds were inoculated with either *M. salina* (CCMP 1776, formerly *Nannochloropsis salina*; ARW4, 6, 9, & 11) or *P. tricornutum* (“Flour Bluff” isolate; ARW1, 5, 7, and 12) using natural, diatomaceous earth filtered seawater from Laguna Madre, Corpus Christi, TX, on 27 October 2015 (Fig. S1). Cultures were amended with 2.0 mM NH_4_Cl, 2.0 mM pH-balanced H_3_PO_4_, and 0.07 mM FeSO_4_. The first harvest (6 November 2015) removed 75% of the cultures with media replacement and was followed by 50% harvests (with media replacement) on 10, 13, 17, 20, and 24 November. Samples for ash-free dry weight (AFDW) were collected on 27 October (day 1), 2 November (day 7), 11 November (day 16), 17 November (day 22), 24 November (day 29), 27 November (day 32). Due to visible clearing of *P. tricornutum* biomass, samples for AFDW were only collected for *M. salina* ponds on 2 December (day 37) and 3 December (day 38; Fig. S1). Samples for sequencing were collected from all ponds on days 1, 7, 29, and 38 (Fig. S1). pH, salinity, temperature, and weather station data were collected daily. Each raceway had a pinpoint marine pH controller that operated a solenoid to inject CO_2_ into the raceway through a ceramic airstone based on raceway pH. Water samples were shipped overnight to Lawrence Livermore National Laboratory (LLNL) on blue ice in polycarbonate clear bottles and processed for DNA extraction as outlined below. The remaining live samples were incubated in plant growth chambers under a 14/10 L/D cycle at 200 µmol quanta m^−2^ s^−2^ and 22°C for 1 week. To test whether a member of the bacterial community could be responsible for crashes, a sample from the ARW7 pond from day 32 was filtered with a 0.8 µm syringe filter, and 1 mL of this filtrate was added to a 100 mL flask of a previously axenic *P. tricornutum* CCMP 2561 culture in instant ocean F/2 medium. This flask showed visibly lower algal cell density and a cloudy color after incubation for 1 week, compared to a no-addition flask. This addition was repeated two more times, after which the crashing could no longer be propagated. Because we lost the activity before attempting to cultivate the microbe on standard media, we were unable to isolate the bacterium.

### 16S/18S rRNA gene amplicon sequencing

Overnight shipped samples were immediately filtered onto 0.22 µm pore size SUPOR filters to capture both algal and bacterial biomass and extracted for DNA using the Qiagen DNEasy kit. The 16S and 18S rRNA genes were amplified using 16S V4 primers 515F (31) and 806R (32) and 18S rRNA V4 primers 565F and 908R (33). Amplicons were sequenced through the Joint Genome Institute’s Community Sequencing Program using the Illumina MiSeq (JGI CSP). DADA2 v1.6.0 (34) was used for quality control, and removeBimeraDenovo was used to remove chimeras. Silva v138 (35) was used to assign taxonomy to the 18S/16S amplicon sequence variants (ASVs). The 16S ASVs were further assigned with the RDP classifier v2.11 (36) against training set 16.

### Metagenomes

To generate a library of metagenome-assembled genomes (MAGs) in the ponds, total DNA extracted from two out of four *P. tricornutum* ponds (ARW1 and ARW7) was submitted for metagenome sequencing through JGI’s CSP with the Illumina NovaSeq S4 (Fig. S1). Raw reads were quality controlled using the JGI Standard Operating Procedure (37). Two co-assemblies were generated using QC’d reads across each pond using metaSPAdes v3.13.0 with kmers 21, 33, and 55 (38). Binning was done inside metawrap v1.3 (39) with metabat v2.12.1, maxbin v2.2.6, and concoct v1.0.0. Bins were assessed with checkM v1.1.3 (40), and those with a completion >50% and contamination <10% were kept and reassembled as individual assemblies using SPAdes v3.13.0 (41). Resultant MAGs were dereplicated into a set of non-redundant MAGs using dRep v2.2.3 (42). To estimate MAG relative abundances, QC’d reads were mapped to the MAGs with bbmap v35.85 (43) and perfectmode = t. MAG abundance was calculated using the mean of the median fold coverage (“MAG abundance”) in each sample (44).

MAG taxonomy was assigned with GTDB-tk v.1.0.0 r202 using default parameters (45). The PATRIC pipeline (46, 47) was used for gene calling and functional annotation. GATOR (https://github.com/jeffkimbrel/gator) was used in parallel to assess metabolic pathway completeness, curated for pathways relevant to algal microbiomes and unique carbon/energy utilization pathways. Carbohydrate-active enzyme (CAZyme) Hidden Markov models (HMMs) were queried using dbcan12 with dbCAN2 (48). Orthofinder 2.5.5 (49–51) was used to find orthogroups, and a species tree was generated using default parameters and annotated using iTol v5 (52). Genomes for 6 *Kordia* species (*Kordia algicida* OT-1 [GCF_000154725.1], *Kordia periserrulae* [GCF_003054265.1], *Kordia antarctica* [GCF_009901525.1], *Kordia jejudonensis* [GCF_001005315.1], *Kordia zhangzhouensis* [GCF_001005305.1], and *Kordia* sp. SMS9 [GCF_003352465.1]) were downloaded from NCBI. Amino acid sequences of these strains and our *Kordia* sp. MAG (“mARW1_16”) were clustered with Orthofinder v2.3.11 (49) to obtain a species phylogenomic tree. Average nucleotide identity (ANI) was calculated on the genome sequences using FastANI v1.3 (53).

### Metaproteomes

Total proteins were extracted following Mayali et al. (11). A Waters nano-Acquity dual pumping UPLC system (Milford, MA) was configured for online trapping of a 5 µL injection at 5 µL/min with reverse-flow elution onto the analytical column at 300 nL/min. MS analysis was performed using a Q-Exactive HF mass spectrometer (Thermo Scientific, San Jose, CA) outfitted with a home-made nano-electrospray ionization interface (see Supplemental methods for more detail). Protein spectra were mapped to a database of assembled MAGs (1,134,297 sequences), a *P. tricornutum* genome [10,408 sequences, *Phaeodactylum tricornutum* CCAP1055.1 (54)], public *M. salina*/*Nannochloropsis* genomes (10,964 sequences, NCBI TaxID 5748) obtained in March 2021, and 16 common contaminant proteins. Sequences were processed using Protein Digestion Simulator (https://github.com/PNNL-Comp-Mass-Spec/Protein-Digestion-Simulator). MSGF+ (55) was used to identify peptides against the custom protein database in target/decoy mode with 20 ppm parent ion tolerance, tryptic rule without post-translational modifications considered. Best MSGF+ search matches were filtered at 1% FDR, and MASIC (56) was used to pull identified peptide abundances. Only protein-specific peptides (peptides unique to the protein in the whole protein collection) were used in subsequent analysis and aggregation. Protein abundance is reported as normalized spectral abundance factor (NSAF) as described in reference 57. The *Kordia* sp. (MAG “mARW1_16”) expressed proteins were manually assigned KEGG functional categories using the PATRIC gene calls. GATOR pathways were merged with this set of proteins, and data were plotted using ggplot2 (58).

### Cross-feeding laboratory experiment

We tested whether pre-conditioning of *P. tricornutum* lysate (analogous to demise conditions) by *Kordia algicida* OT-1, as the primary degrader, enhanced the subsequent growth of a representative *Rhodobacteraceae* strain (*Sulfitobacter* sp. N5S). OT-1 was purchased from the Japanese National Institute of Technology and Evaluation (NITE) Biological Resource Center (NBRC Accession #100033). We annotated the public genome as described above. Strain N5S was isolated from Bodega Bay, CA (38.332615, –123.048296) by spreading whole seawater onto marine agar plates, and its genome was sequenced by JGI (NCBI Accession # PRJNA581036). See the Supplemental Methods for details.

*P. tricornutum* CCMP 2561 lysate was generated using an axenic exponentially growing culture in sterile F/2 media as described in Supplemental Methods (axenicity checked with DAPI staining and fluorescence microscopy). Algal cells were pelleted by spinning at 4,500 × *g* for 8 min, and the spent media discarded to remove algal exudate. Cells resuspended in sterile F/2 were lysed using an Ultrasonic Processer XL sonicator (Misonix) and filtered through a 0.8 µm syringe filter to remove intact cells. The final lysate was then incubated with or without *K. algicida* to produce “abiotic” and *Kordia* “conditioned” lysate. This experiment was carried out in triplicate in 3 mL sterile borosilicate tubes at 22°C in the dark for 5 days. Growth was monitored using OD_600_ (BioTek Cytation 5 plate reader, Agilent) and flow cytometry. Strains OT-1 and N5S were then inoculated into 0.2 µm filtered abiotic versus conditioned lysate in five replicates in a 96-well plate (rounded bottom). Negative controls (no OT-1 and N5S bacteria added) and a positive control (high-nutrient Zobell media) were included. OD_600_ absorbance was collected every 20 min for 72 h using a BioTek Cytation 5 plate reader at 22°C in the dark. Flow cytometry samples were collected at *T* = 0 h and *T* = 72 h.

### Statistical methods

The R software platform (59) was used for statistics and data visualization (ggplot2 [58]). Differential abundance of ASVs comparing day 1 versus day 38 was done with ANCOM-BC (v.2.2.2) (60). The VEGAN (v.2.6-4) (61)) package was used for the following: nonmetric multidimensional scaling (NMDS) based on Bray-Curtis distances, adonis function for significance of clusters, and diversity function for Shannon and Simpson’s indices. To compare normalized protein abundance across time, an ordinary one-way ANOVA followed by the Benjamini-Hochberg adaptive false discovery rate (FDR) procedure to correct for multiple comparisons was conducted using GraphPad Prism 9. For the conditioning experiment, unpaired two-tailed *t*-tests were used to compare cell abundances at *T* = 72 h using GraphPad Prism 9. Growth rates were calculated on linear regressions of exponential phase growth (OD_600_) using GraphPad Prism 9.

## RESULTS

### Differential persistence of the two algal species and evidence of *P. tricornutum* population decline in the outdoor algal raceway ponds

During concurrent cultivation of 8 co-located ARW ponds (four inoculated with *Phaeodactylum tricornutum*, and four inoculated with *Microchloropsis salina*), an algal crash occurred only in the four *P. tricornutum* ponds after 29 days of semi-continuous cultivation. A visible change in color from dark brown (healthy *P. tricornutum* biomass) to a milky white was noted. A steady decline in algal biomass (~0.3 to <0.1 g/L) across the *P. tricornutum* ponds occurred (Fig. S2; Table S1), but not for *M. salina* after day 29 (Fig. S2). During cultivation, pond salinity ranged from 31 to 35 ppt, pH from 7.38 to 8.6, and water temperature from 18.0°C to 28.0°C (Table S2).

**Fig 1:**
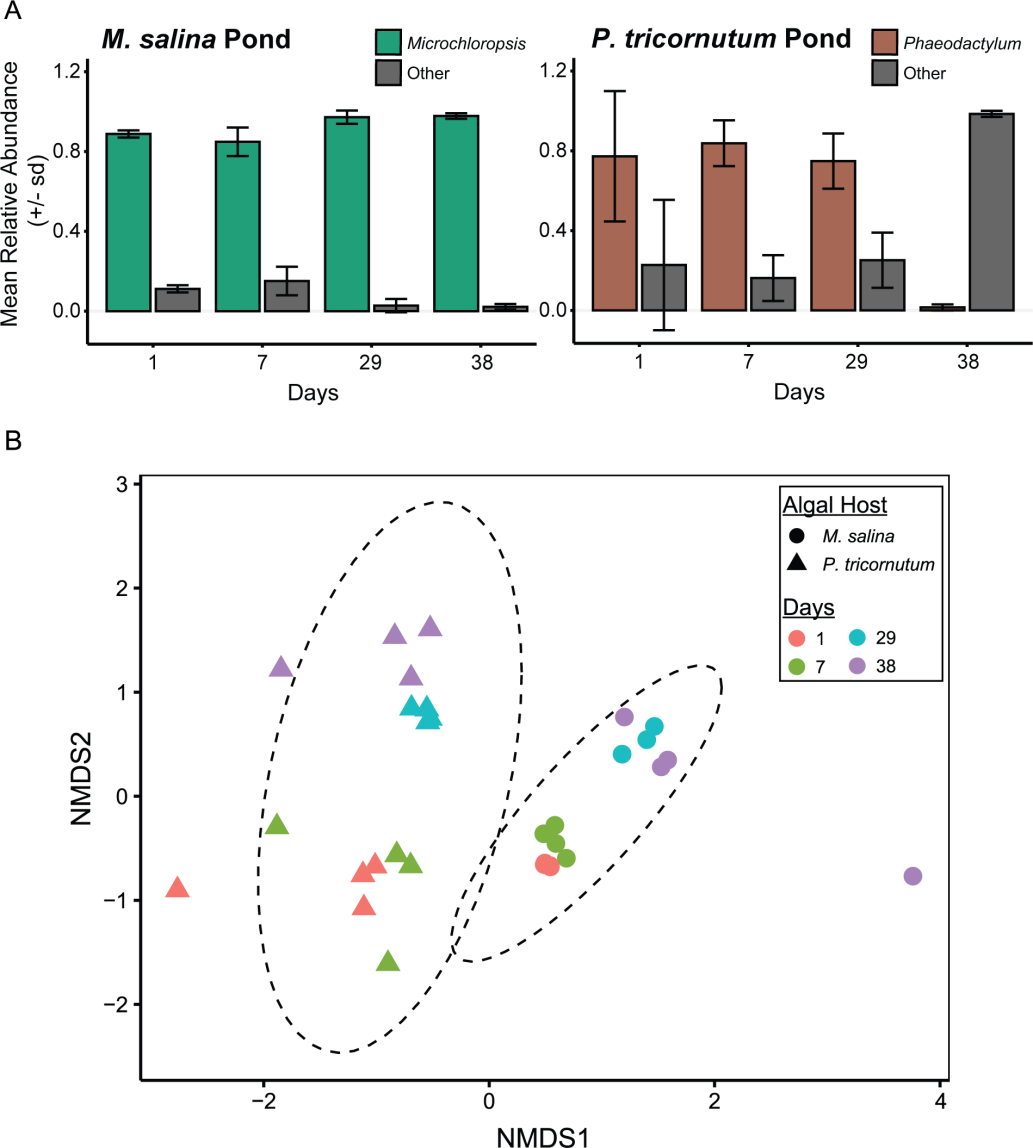
Algal host strain and decline in *P. tricornutum* over time influence microbiome composition within the algal raceway ponds. (A) Relative abundance of representative host 18S rRNA ASVs between ponds inoculated with *M. salina* versus *P. tricornutum*. Error bars represent the standard deviation in relative abundance between four replicate ponds. 18S rRNA amplicons assigned to either *Phaeodactylum* or *Microchloropsis* are shown in either brown or green, where all other 18S rRNA amplicons are shown as “Other” in gray. (B) NMDS ordination based on the Bray-Curtis dissimilarity matrix of 16S rRNA bacterial communities between samples inoculated with *M. salina* (circles) and *P. tricornutum* (triangles) over timepoint (indicated by color); 95% confidence ellipses (stat_ellipse) are grouped by algal host inoculum. Stress = 0.115.

To further quantify *P. tricornutum* demise, we used 18S rRNA gene profiling, focusing on the genera assignments *Microchloropsis* or *Phaeodactylum* within their respective ponds. *Microchloropsis* ponds exhibited stable host 18S rRNA relative abundance, whereas *P. tricornutum* ponds significantly declined in the relative abundance of host 18S rRNA at day 38 (5.8-log fold change between day 1 versus day 38 [ANCOMBC, *P*_adj_ = 0.0001]; Fig. 1A; Table S3). Proteomics also indicated a declining trend in proteins identified as *P. tricornutum* on days 29 and 38, with negligible proteins identified as *M. salina* (Fig. S3). Therefore, we refer to this event as a population-wide “crash,” with “demise” referring to the steady decline in ash-free dry weight of *P. tricornutum* after day 16 (Fig. S2).

### Bacterial community composition responded to algal host and health

Each algal host had distinct microbiome compositions that shifted with time (Fig. 1B), especially pronounced for *P. tricornutum* ponds (Fig. S4; presumably due to demise). NMDS ordination clustered 16S rRNA gene microbiome profiles based on algal host (Fig. 1B; ADONIS *P* < 0.0001). The dominant bacterial groups differed between the host alga (Fig. S5). *M. salina* ponds were dominated by ASVs assigned to the *Alpha*- and *Betaproteobacteria*, *Sphingobacteriia*, and “unclassified” *Pseudomonadota* (Fig. S5). The *P. tricornutum* ponds were dominated by ASVs assigned to the *Alpha*-, *Gamma*-, and *Betaproteobacteria* at days 1 and 7 but shifted to predominantly *Flavobacteriia* and *Alphaproteobacteria* classes at days 29 and 38 (Fig. S5). We focused further analyses on the *P. tricornutum* pond microbiome due to evidence of algal population demise. Based on NMDS clustering, two major clusters of ASVs in the *P. tricornutum* microbiome emerged (Fig. S4a; ADONIS *P* < 0.005). These clusters corresponded to *P. tricornutum* growth phases that occurred in the four replicated ARW ponds: “growth” (days 1 and 7) and “demise” (days 29 and 38). The alpha-diversity of the demise microbiome was significantly reduced compared to the growth microbiome (Fig. S4B).

### Taxon-specific response to *P. tricornutum* demise—identification of a highly abundant bacterium, *Kordia* sp.

**Fig 2:**
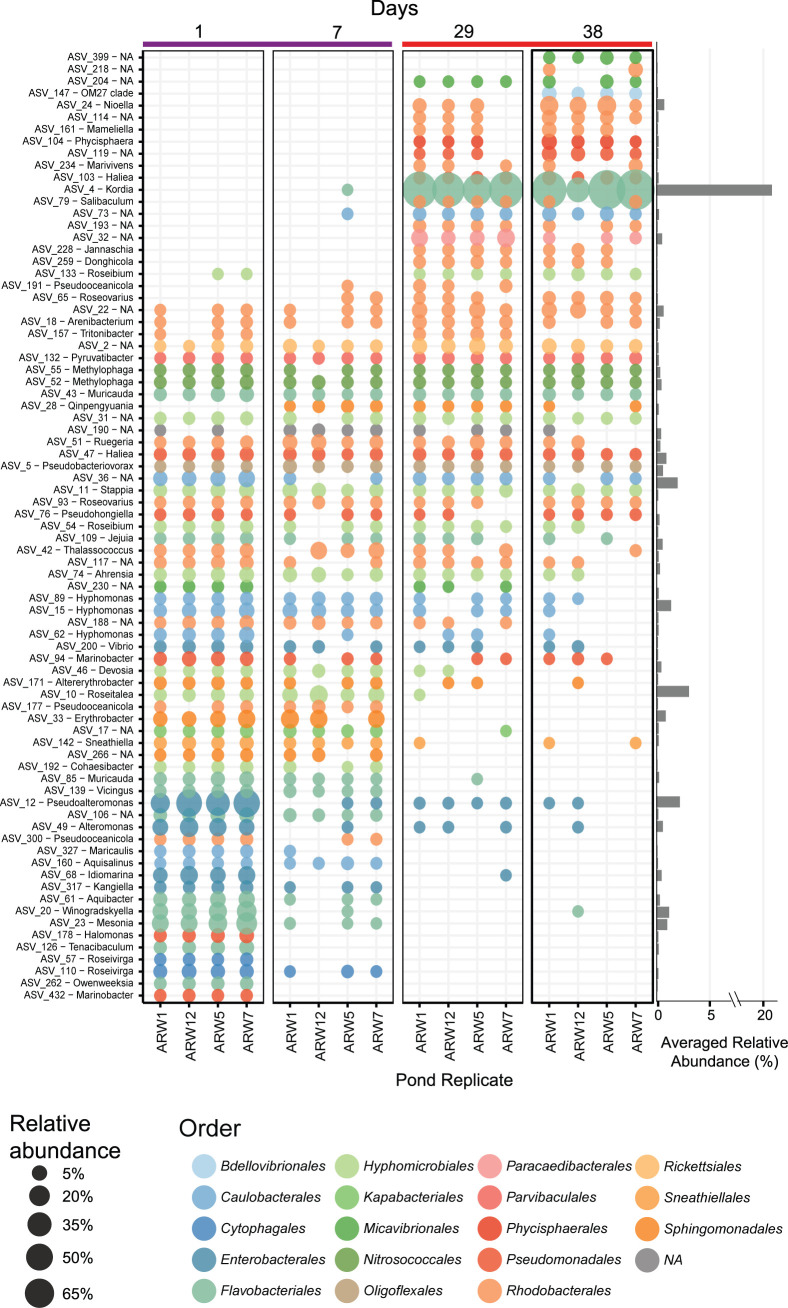
Shift in microbiome composition during *P. tricornutum* demise corresponds to a bloom of an individual ASV assigned to the genus, *Kordia*, and co-occurring taxa. Each pond replicate is shown on the *x*-axis, and individual ASVs (shown with ASV number and lowest taxonomic assignment) are on the *y*-axis. The timepoints are grouped and color-coded by algal growth phase, which is based on NMDS clustering of the relative abundance of ASVs in Fig. S4 (growth [purple line] versus demise [orange line]). Each bubble represents the relative abundance value for each replicate, and the averaged relative abundance across all the plotted samples for each ASV is shown on the bar graph to the right. Bubbles are color-coded by the taxonomic assignment to the order level according to the SILVA v138 database.

Examination of individual ASVs across the *P. tricornutum* growth phases revealed an ASV-specific succession (Fig. 2). One ASV assigned to the genus *Kordia* (ASV_4) dominated the demise phase (reaching up to 93% relative abundance of the 16S rRNA reads, Fig. 2), and increased 12.5-log fold on day 38 versus day 1 (ANCOMBC, *P*_adj_ = 3.47e−75, Table S4). ASV_4 was nearly undetectable during growth timepoints (Fig. 2) and had no detectable reads in the *M. salina* microbiome (Fig. S6). Aside from ASV_4, the demise microbiome was primarily composed of many ASVs assigned to the order *Rhodobacterales*, with ASV_24 (*Nioella* sp.) reaching up to ~6% relative abundance within a sample (Fig. 2). By contrast, the growth microbiome had greater diversity of order-level taxonomic affiliations, with non-*Kordia Flavobacteriales*, *Enterobacterales, Cytophagales,* and *Sphingomonadales* contributing high relative abundances (Fig. 2). Several *Rhodobacterales-*, *Hyphomicrobiales-,* and *Nitrosococcales-*assigned ASVs persisted throughout both phases (Fig. 2).

**Fig 3:**
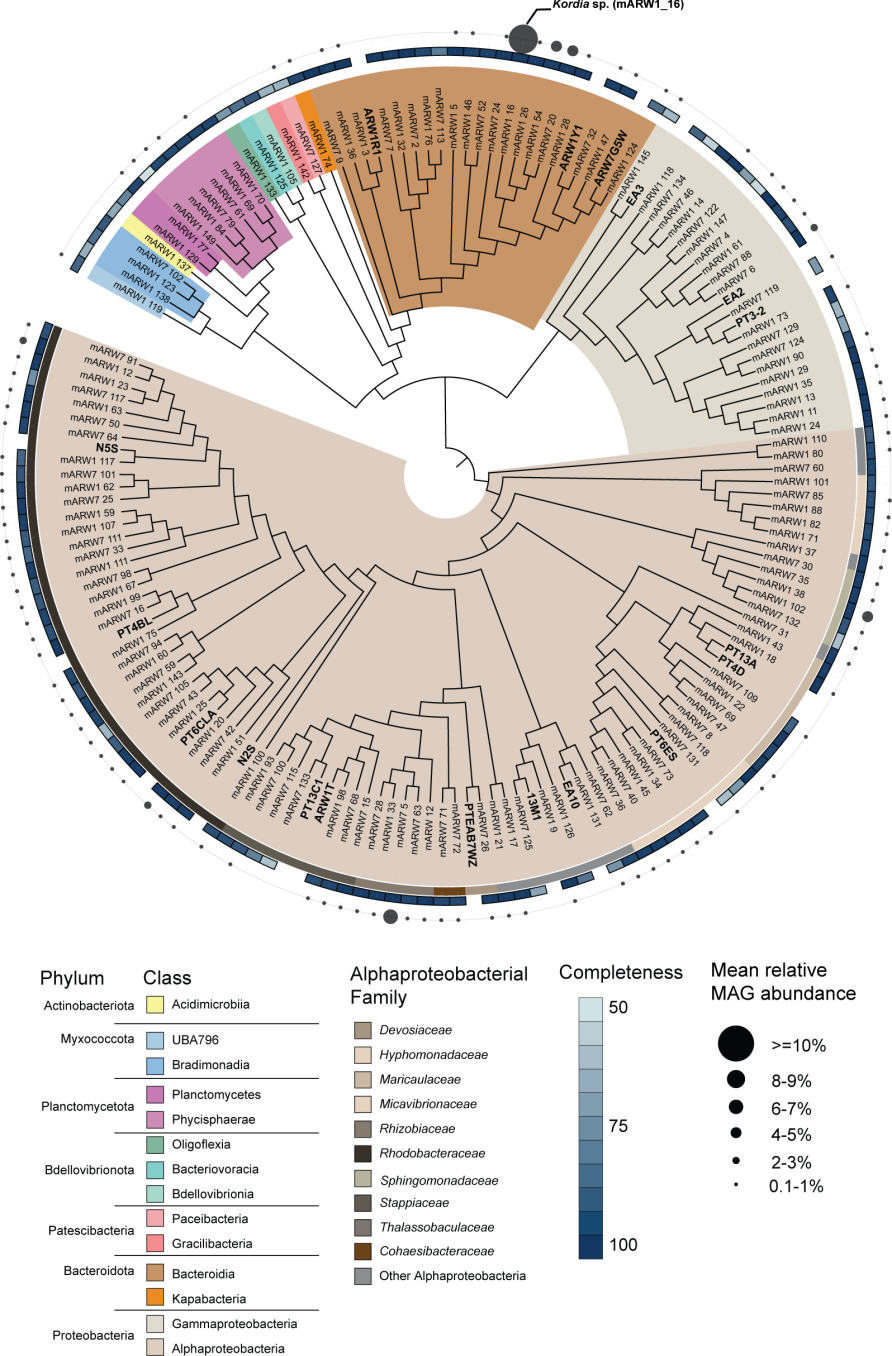
Distribution of MAGs detected in the *P. tricornutum* pond metagenomes. The phylogenetic tree shows the relationship between the MAGs based on orthogroups (see Materials and Methods). Cultivated isolates are denoted by their strain identification around the tree as follows: PT13A: *Oceanicaulis* PT13A, ARW1R1: *Algoriphagus* ARW1R1, ARW1Y1: *Muricauda* ARW1Y1, ARW7G5Y1: *Arenibacter* ARW7G5Y1, PTEAB7WZ*: Devosia* PTEAB7WZ, ARW1T: *Stappia* ARW1T, PT6CLA: *Rhodophyticola* PT6CLA, PT4BL: *Yoonia* PT4BL, EA3: *Pusillimonas* EA3, EA2: *Alcanivorax* EA2, PT6ES: *Henriciella* PT6ES, EA10: *Tepidicaulis* EA10, PT13C1: *Roseibium* PT13C1, 11-3: *Thalassospira* 11-3, PT3-2: *Marinobacter* 3-2, ARW7G5W: *Muricauda* ARW7G5W, PT4D: *Oceanicaulis* PT4D, N2S: *Roseobacter* N2S, N5S: *Sulfitobacter* N5S. The relative abundance range of MAGs averaged across all samples is shown as bubbles surrounding the tree. MAG completeness shown as a heatmap ranging from 50% to 100% completeness. Clades are color-coded by Class-level GTDB taxonomy, and to the Family level for Alphaproteobacteria.

MAGs were generated for two of four raceways from the *P. tricornutum* ponds (“ARW1” and “ARW7”, Table S5). A total of 143 non-redundant MAGs were assembled, some of which cluster with bacterial strains previously isolated from these ponds and co-cultured with *P. tricornutum* (11, 62) (Fig. 3). The phyla *Myxococcota, Planctomycetes, Bdellovibrionota*, *Patescibacteria*, *Bacteroidota*, *Gammaproteobacteria,* and *Alphaproteobacteria* were also detected (Fig. 3). Within the *Alphaproteobacteria*, there was high representation (33 MAGs) of the *Rhodobacteraceae* (Fig. 3). The *Kordia* MAG (mARW1_16, Fig. 3) had the highest MAG abundance values across the *P. tricornutum* ponds (average 28% relative abundance, Fig. 3). We also assessed coverage of metagenome reads mapped to the MAGs, with the caveat that these were only done in duplicate. However, like the amplicon data, *Flavobacteriaceae* and *Rhodobacteraceae* family-level assignments were the dominant groups during demise, whereas the growth phase had greater diversity (Fig. S7). However, MAGs of the *Pirellulaceae, Rhizobiaceae,* and *Phycisphaeraceae* families were also present in the demise phase in lower abundances (Fig. S7).

Members of the *Kordia* genus have been implicated in the demise of marine harmful algal blooms (63, 64), and the *Kordia* MAG was similar to a known algicidal *Kordia*-sequenced genome (*K. algicida* OT-1; Fig. S8) with 2,996 shared orthologs and an average nucleotide identity of 81% (65). Both the OT-1 genomes and the *Kordia* MAG include a protease previously hypothesized to be involved in algicidal activity (29). Although we lack direct evidence that *Kordia* was responsible for *P. tricornutum's* demise, it is reasonable to hypothesize that it played a role in this process. One indication is that a single genotype dominated the microbial community prior to and during the diatom collapse, similar to other bacterial pathogens (66, 67). In addition, previous investigations have implicated this genus in the demise of natural algal blooms, both through laboratory experiments and quantification throughout the bloom cycle (30). Furthermore, at the time of sampling the ponds, we were able to propagate algicidal activity from a live and 0.8 µm filtered ARW7 sample into previously axenic *P. tricornutum* cultures, suggesting a bacterial component could recreate an algal culture crash. We were not successful in maintaining this algicidal community and thus could not directly attribute *P. tricornutum* death to *Kordia*.

### Expressed proteins mapped to *Kordia* sp. provide insight into mechanisms leading to its dominance during algal pond demise

**Fig 4:**
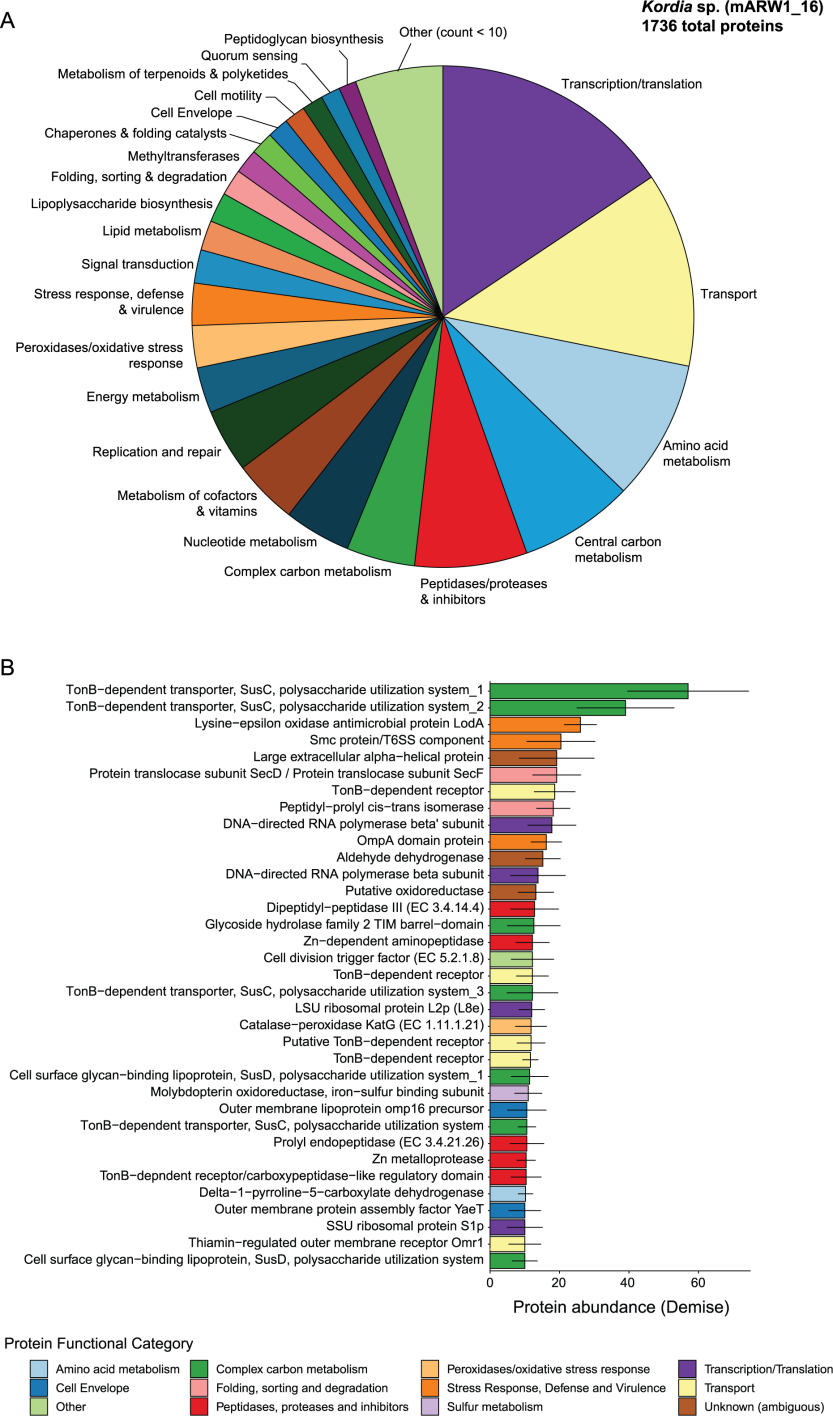
Abundant protein functional categories and proteins mapped to the *Kordia* MAG indicate antagonism and biopolymer degradation. (A) Breakdown of the most detected protein functional categories mapped to *Kordia*. The "Other" category specifies categories with less than 10 detected proteins across all metaproteomics samples. Proteins with "unknown" or "ambiguous" function were excluded for visual purposes. Protein assignments are available in Table S6. (B) The topmost abundant *Kordia* proteins detected across the demise samples (days 29 and 38) that had an average NSAF ≥ 10. Average NSAF across all demise samples (*n* = 5) is shown, and their standard deviations are represented as error bars.

Using metaproteomics of *P. tricornutum* pond samples over time, we identified expressed proteins mapping to the *Kordia* MAG (mARW1_16) to gain insight into the metabolic activity of *Kordia* during diatom demise. There were 1,728 of 4,252 (40.64%) proteins in the mARW1_16 MAG detected in the metaproteome (Fig. S9; Table S6). Proteins in the Transcription/Translation, Transport, Amino acid metabolism, and Central carbon metabolism categories were most detected, suggesting the *Kordia* population was metabolically active (Fig. 4A). The next most abundant categories were the Peptidases/proteases & inhibitors and Complex carbon metabolism (Fig. 4A). Furthermore, the Complex carbon metabolism category harbors the most abundant protein detected (based on normalized spectral abundance factor [NSAF]) across demise/crash samples: a SusC-family Ton-B-dependent transporter (Fig. 4B). The gene encoding this protein is located nearby genes encoding glycoside hydrolase family 2 TIM barrel-domain, beta-glucanase, and a putative lamarinase targeting beta-glucan, all detected in the *Kordia* proteome (Table S7). Likewise, several proteins assigned as SusD surface glycan-binding PUL component (Complex carbon metabolism) were abundant (Fig. 4B).

The next two most abundant proteins belonged to stress response, defense, and virulence (Fig. 4B). The lysine-epsilon oxidase protein (LodA), known for its antimicrobial activity resulting from hydrogen peroxide production (68), was among the top three most abundant *Kordia* proteins expressed across algal demise (Fig. 4B; Table S6). In addition, a putative type VI secretion system (T6SS) component was the fourth most abundant protein (Fig. 4B) and is located nearby two expressed T6SS baseplate protein components (TssJ and VgrG) and many other encoded T6SS components (Table S6). It is believed that algicidal *Kordia* members secrete a protease involved in algal killing (29); thus, we searched for this protein in the proteome. This protein was detected in five samples, all of which were in the demise phase (Table S6). However, it was detected in low abundance (total spectral counts of 23, Table S6), potentially because we only sampled the intracellular proteome, missing the extracellular fraction where it is most likely to be detected.

### Genomic analysis indicates *Kordia* fills a specific niche compared to the co-occurring *Rhodobacteraceae* community

**Fig 5:**
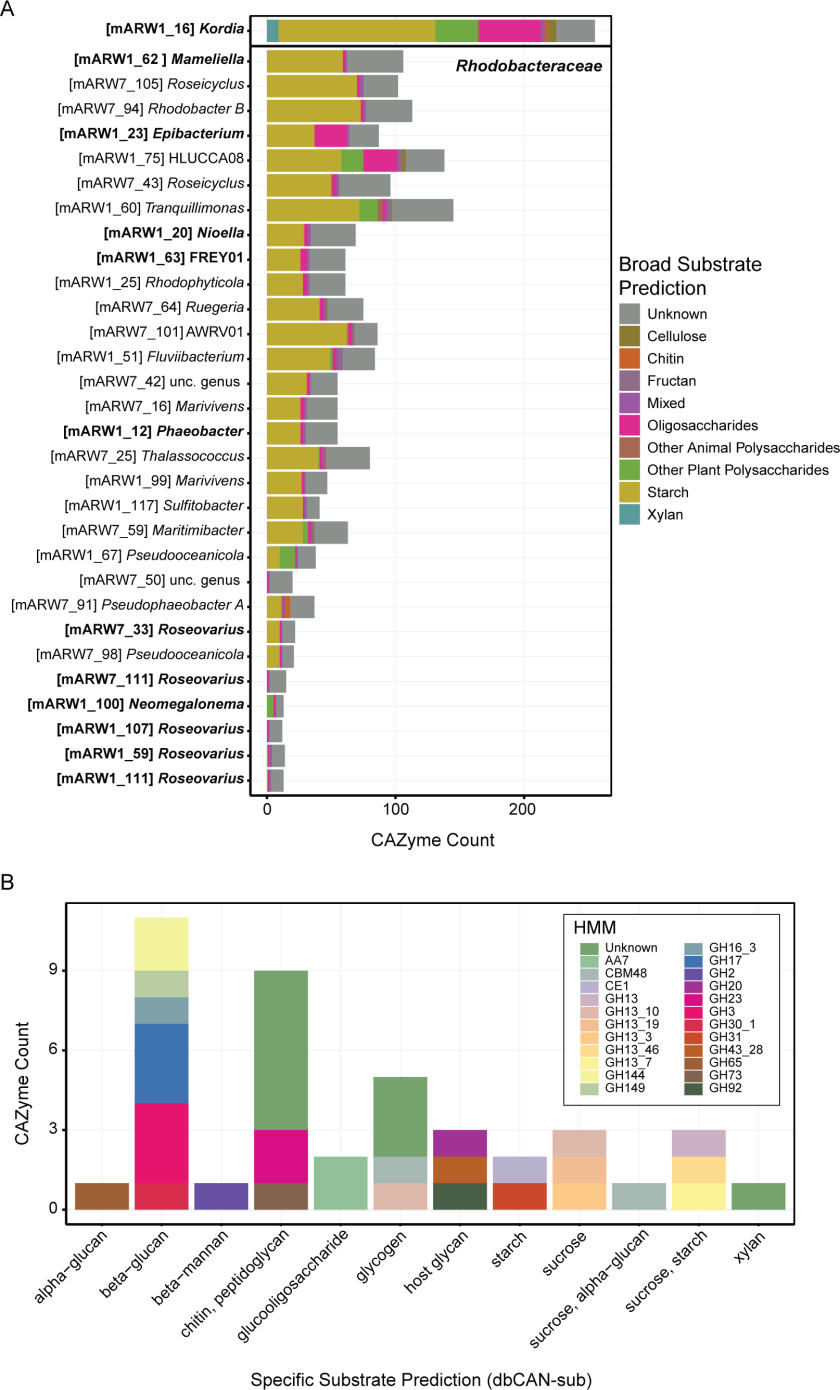
*Kordia* fills a distinct niche compared to *Rhodobacteraceae* during algal demise by encoding abundant CAZymes targeting a diverse range of predicted biopolymers. (**A**) All *Rhodobacteraceae* non-redundant MAGs at ≥75% completion (*n* = 30) assembled from either the ARW1 or ARW7 ponds are shown for comparison with the *Kordia* MAG (boldened). MAG IDs are shown in brackets, followed by their genus-level assignment. Only CAZyme hits to glycoside hydrolases (“GH”) or polysaccharide lyases (“PL”) are included in the summarized CAZyme counts per MAG, and are color-coded by their predicted substrate. Emboldened MAGs indicate taxa that were active during the demise phase, as indicated by metaproteomics (see Fig. S9). (**B**) Specific CAZyme HMM hits and dbCAN substrate annotations for *Kordia* (mARW1_16). Hits with no substrate predicted (*n* = 81) were removed for visual purposes.

We initially hypothesized that microbial taxa present during the demise phase may have metabolized complex biomolecules expected to be released from dead or dying diatom cells. We then asked whether taxa in the demise microbiome, other than *Kordia* sp., were specialized to acquire carbon from biopolymers. We examined the CAZyme profiles of *P. tricornutum* pond MAGs, focusing on comparing the *Kordia* sp. MAG to all MAGs assigned to the *Rhodobacteraceae* family, as these two groups dominated the demise microbiome (Fig. 2; Fig. S7). We counted all CAZyme hits to glycoside hydrolases (GH) and polysaccharide lyases (PL) and summarized them at the individual MAG level (Fig. 5A). We found that *Kordia* sp. harbored ~255 of these CAZymes, while the *Rhodobacteraceae* MAGs averaged ~61 CAZymes per MAG (*n* = 30 MAGs; Fig. 5A). In the *Kordia* MAG specifically, CAZymes were predicted to target a diverse range of substrates such as beta-glucan (>10), chitin/peptidoglycan (>7), glycogen (5), and several others (Fig. 5B). Many of these were also detected abundantly in the metaproteome during the demise phase (Table S8).

Some *Rhodobacteraceae* MAGs harbored a diverse repertoire of CAZymes (e.g.*,* mARW1_75, mARW1_60); however, MAGs with specific genus-level matches to the ASVs grouped in the demise phase were relatively depleted in diverse CAZymes (Fig. 5A). Furthermore, we did not find many *Rhodobacteraceae* CAZymes expressed in the proteome (spectral count ≤4 in a given sample; Table S8). Those *Rhodobacteraceae* CAZymes with the highest total spectral counts were found during the growth phase timepoints (Table S8). By contrast, the most abundantly detected proteins belonging to *Rhodobacteraceae* MAGs during the demise phase were transporters for sugars and amino acids, such as a glycerol ABC transporter substrate-binding component (mARW1_12.peg.2571), glutamate/glutamine/aspartate/asparagine ABC transporter substrate-binding component (mARW1_23.peg.3083), TRAP-type C4-dicarboxylate transport system periplasmic component (mARW1_20.peg.1354, mARW1_12.peg.44), and a fructose ABC transporter substrate-binding component (mARW1_23.peg.3255, Table S6).

### *Sulfitobacter,* an ecologically relevant Roseobacter-clade member, required *Kordia* to process *P. tricornutum* lysate

**Fig 6:**
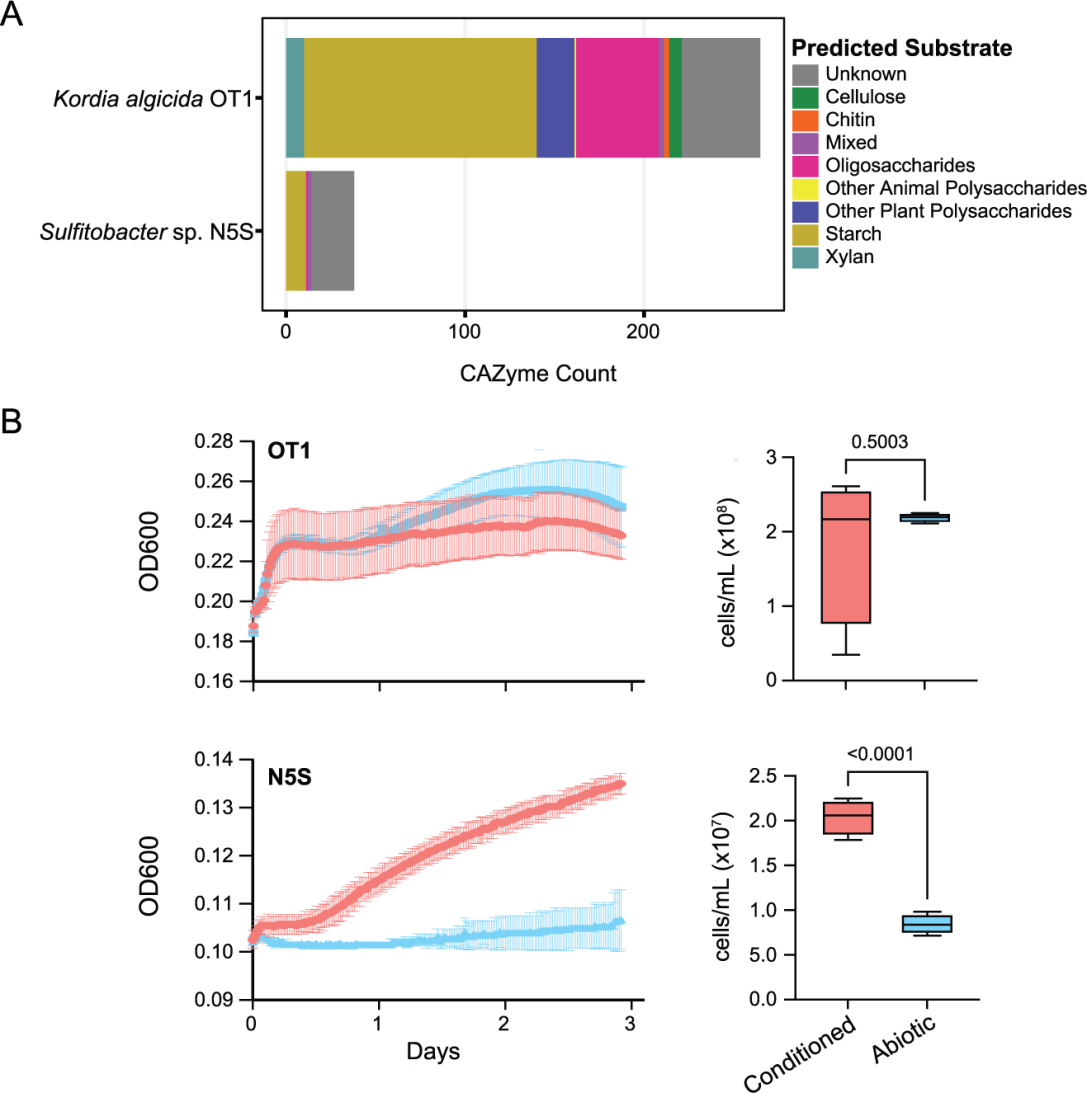
*Sulfitobacter* sp. N5S (a representative *Rhodobacteraceae* strain) requires *Kordia* to pre-condition *P. tricornutum* lysate for growth in culture. (A) CAZyme summary of representative lab strains of *Kordia* (*K. algicida* OT1) and a *Rhodobacteraceae* (*Sulfitobacter sp*. N5S) used for the experiment. (B) Growth of OT1 versus N5S on *P. tricornutum* lysate either pre-conditioned by OT-1 (“Conditioned”) or without any added bacteria (“Abiotic” control). Growth curves show the mean and standard deviation (error bars, *n* = 5) of OD_600_ values taken every 20 minutes. The boxplots to the right show the cell abundances at the final timepoint (*n* = 4). *P*-values are generated using unpaired two-tailed *t*-tests.

As *Rhodobacteraceae* taxa present in the demise microbiome generally lacked the capability to degrade complex carbon sources, we hypothesized that the activity of *Kordia* sp., a specialized biopolymer degrader, might allow for the growth of these small-molecule users during diatom demise. To test this, we performed a laboratory experiment using a representative *Kordia* strain (*K. algicida* OT-1) and an isolate of the *Rhodobacteraceae* family (*Sulfitobacter* sp. N5S). This *Rhodobacteraceae* strain was selected because it is related to relatively abundant MAGs (*Sulfitobacter* sp. [mARW1_117] and *Phaeobacter* sp. [mARW1_12]; Fig. 3) in the *P. tricornutum* ponds and could be classified as a strict small molecule user due to a lack of CAZymes in its genome (Fig. 6A). Furthermore, this genus falls within the Marine Roseobacter Clade, an ecologically relevant and ubiquitous group found in marine environments (69, 70). This isolate also grows in co-culture (growth rate of 0.35 day^−1^) with *P. tricornutum* (growth rate of 0.35 day^−1^) at perpetuity with no added organic matter (Fig. S11).

*K. algicida* OT-1 has been shown to have algicidal activity toward *P. tricornutum* when grown in high-nutrient media (29) and harbors a diverse repertoire of CAZymes targeting complex carbon sources (Fig. 6A). We cultivated *K. algicida* strain OT-1 in *P. tricornutum* lysate, an analog to the demise phase of the algal ponds, for 5 days. Then, we filter-sterilized the spent media (“conditioned” lysate) to remove *Kordia* cells and inoculated this conditioned lysate with *Sulfitobacter* strain N5S. There was a significantly (*P* < 0.0001; Table S9) reduced growth rate of *Sulfitobacter* N5S in the abiotic lysate (0.02 day^−1^) compared to its growth rate in conditioned lysate (0.09 day^−1^), with significantly different final cell abundances between the treatments (*P* < 0.0001; Fig. 6B). *K. algicida* did not have significantly different (*P* = 0.0614; Table S9) growth rates in both its own conditioned lysate (0.10 day^−1^) and abiotic lysate (0.08 day^−1^) with no significant difference in the final cell abundances (*P* = 0.5003; Fig. 6B). This suggests *Sulfitobacter* was dependent on *Kordia* to access the organic matter present in *P. tricornutum* lysate. Furthermore, the finding that *Kordia* did not grow differentially under conditioned versus abiotic lysate suggests that the algal-derived carbon was not fully metabolized during the first incubation, and *Kordia* limited its growth through an unknown mechanism.

### Genomic capability to metabolize alternative compounds in marine *Rhodobacteraceae*

**Fig 7:**
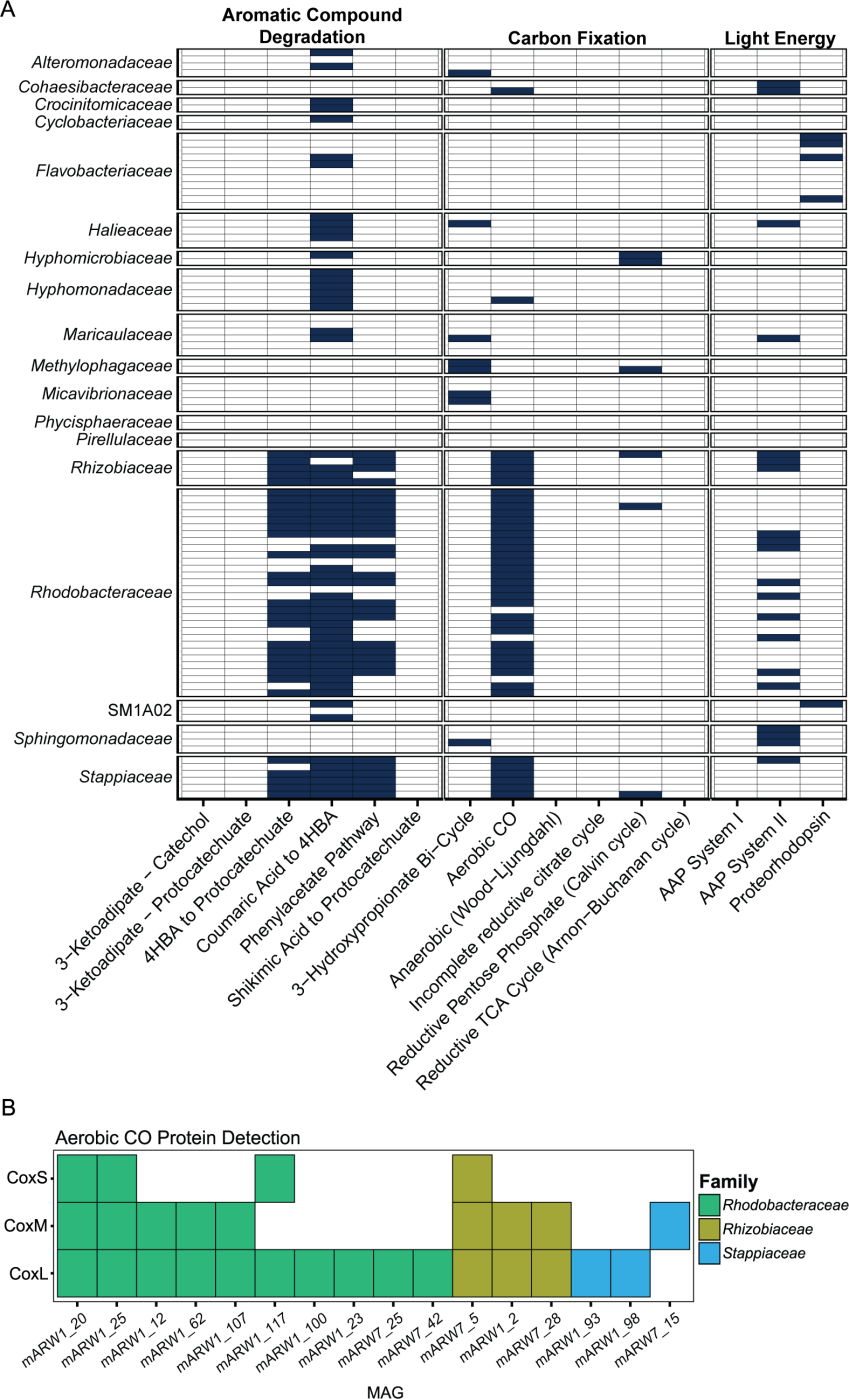
Carbon monoxide (CO) oxidation as a potential alternative carbon and energy-generating mechanism used by *Rhodobacteraceae* in algal ponds. (A) Metabolic potential across all *P. tricornutum* pond MAGs to use alternative carbon and energy sources. All screened pathways are shown (*x*-axis), where dark blue tiles indicate 100% of pathway detection (i.e., all known genes involved in that pathway were detected) per-MAG (*y*-axis, grouped by family). (B) Protein presence/absence of each CODH subunit across MAGs in the metaproteome. Only MAGs that had a detected protein mapped to CODH out of 13 samples are shown.

Members of the *Rhodobacteraceae* family are globally abundant in the oceans and well known for having the ability to rapidly respond to increased substrate availability and acquire carbon and energy from diverse sources (69, 71, 72). We hypothesized that the prevalence of the *Rhodobacteraceae* across the algal growth phases is due, in part, to their ability to grow on diverse carbon and energy sources that are typically not available to other microbes when preferred resources (e.g., sugars and amino acids) are rapidly consumed during competition. Thus, we screened all MAGs from the *P. tricornutum* ponds for pathways that confer the ability to utilize aromatic carbohydrates, fix inorganic carbon, and gain energy from light (Fig. 7). We found that the ability to degrade aromatic compounds such as 4-hydroxybenzoic acid (4HBA), coumaric acid, and phenylacetic acid was common, as was aerobic anoxygenic phototrophy for light-driven metabolism (Fig. 7A). However, 90%–100% of the MAGs in the *Rhodobacteraceae*, *Rhizobiaceae,* and *Stappiaceae* families contained the complete set of genes for carbon monoxide dehydrogenase (COX; *coxL + coxM + coxS*). Moreover, we detected protein expression in the metaproteome of multiple COX subunits across these families, with mARW1_20 (*Nioella sedimensis*), mARW1_25 (unclassified *Rhodobacteraceae*), and mARW7_5 (*Hoeflea* sp.) recruiting all three COX subunits (Fig. 7B). The expression patterns of individual COX proteins across timepoints were variable, especially during the early growth phase (highest expression on day 1; Fig. S12). On the other hand, we detected sparse protein expression of aromatic compound degradation and light-driven metabolism (Fig. S13). This suggests that carbon monoxide utilization was widespread and active in this group, especially during the growth phase.

## DISCUSSION

Our study documents a serendipitous algal population crash that occurred during a multi-omics assessment of outdoor *P. tricornutum* ponds. Initially, we set out to characterize the microbiome dynamics of two algal species cultivated outdoors in pilot-scale biofuel production ponds. Despite close proximity to one another and the open nature of the ponds, we observed distinct microbiomes, shown to be driven by host-specific physiology (73). However, a drop in bacterial diversity during *P. tricornutum* demise corresponded with a “bloom” of *Kordia*, a genus belonging to the phylum *Bacteroidetes* and the *Flavobacteriaceae* family. The *Kordia* genus has been readily cultured from a wide range of aquatic environments and exhibits significant taxonomic and genomic diversity (74, 75). As the *Kordia* sp. reached dominant abundances (up to 93% of the bacterial community), we collected metagenomes and metaproteomes to identify features that may have contributed to its numerical dominance in the outdoor *P. tricornutum* ponds. In addition, we propose that *Kordia* acted as a primary biopolymer degrader, influencing the surrounding microbiota during diatom demise by providing partially degraded products. Specifically, cross-feeding of carbon between biopolymer degraders like *Kordia* (*Flavobacteriaceae*) and copiotrophic *Rhodobacteraceae* may be an ecologically relevant interaction that occurs during algal bloom senescence and die-off.

The genus *Kordia* is primarily known to exhibit an algicidal phenotype against diatom hosts, including *P. tricornutum*, through protease-mediated interactions (29). Genomic analysis of the *Kordia* MAG confirmed the presence of an extracellular metalloprotease putatively involved in algal-specific lysis. However, this protein was not detected in high abundance in the metaproteome (total count of 23 across all demise samples) as only intracellular protein pools were collected due to the sampling procedure that only retained biomass (>0.22 µm). Nevertheless, protease/peptidase-like proteins comprised a major fraction of the *Kordia* proteome during algal demise. These proteins have widely ranging substrate specificities and roles (e.g., antimicrobial, protein folding, biopolymer breakdown, pathogenesis [76]), and particular algicidal proteases could be species-specific, which complicates comparisons with previous studies to validate algicidal activity (77). Although we were able to replicate the diatom crash by incubating the <0.8 µm microbial fraction of crashed ponds with an axenic culture of *P. tricornutum*, we were unable to attempt isolation due to a loss of activity after several transfers. Therefore, although we lack causal evidence that *Kordia* was directly responsible for the diatom pond collapse, no other data suggest other mechanisms. The 18S rRNA gene amplicon data did not identify high abundances of known algal grazers or pathogens such as rotifers, aphelids, or chytrids during the demise phase. Diatom-infecting viruses exist (78), and it is possible that viral lysis was a mechanism of culture demise. Slowly decreasing temperature through the fall season could also have contributed to increased stress in the diatom cells, which could have made them more susceptible to biotic stress.

The metaproteomic data suggest *Kordia* employed mechanisms to inhibit the growth of other microorganisms. One of the most abundantly detected proteins was a putative lysine oxidase (LodA, aka marinocine), a protein shown to be antimicrobial via hydrogen peroxide (H_2_O_2_) produced during amino acid deamination (79). This L-amino acid oxidase hydrolyzes L-amino acids within cells or extracellularly, producing H_2_O_2_, which is bactericidal to a wide range of bacteria (79). LodA-driven H_2_O_2_ production can lead to bactericidal behavior between species under competition and can result in phenotypic variation within biofilm communities (80). Due to the broad-range antimicrobial activity of this oxidase, it is known to also be autotoxic (79). If this process occurred during the *Kordia* bloom, the population was able to withstand the damaging impacts of H_2_O_2_. Indeed, *Kordia* expressed detectable levels of potential detoxification proteins, namely catalase-peroxidase (KatG). The production of these antibacterial pathways suggests that *Kordia* was inhibiting the growth of other bacteria, which is in agreement with the decreased diversity of the microbial community during the algal demise phase. However, these pathways could also have been directly involved in the algal demise, but the literature is lacking on this topic. In addition, we found several type VI secretion system (T6SS) proteins expressed by *Kordia*. T6SS is involved in anti-bacterial and eukaryotic antagonism (81–83), a potential strategy to reduce bacterial competition or gain limiting resources (84). It is possible that *Kordia* employed T6SS activity against *P. tricornutum*; however, T6SS-guided algal killing has not yet been investigated (84).

In addition to antagonism, *Kordia’s* bloom during algal demise may be attributed to its superior ability to access diatom-derived biopolymers, and it simply outgrew its competitors. *Kordia* belongs to the *Flavobacteriaceae* (phylum *Bacteroidota*), a family known to degrade and consume biopolymers in aquatic ecosystems (85, 86). Similar to a previous *Kordia* genomic analysis (74), we found many polysaccharide-degrading CAZymes (87) in the *Kordia* MAG. Furthermore, the most abundant *Kordia* proteins detected were assigned to several polysaccharide utilization loci systems used to extracellularly hydrolyze and import biopolymer carbon using TonB transport systems (24, 86). Although the substrates cannot currently be determined based on sequences alone, these proteins are located upstream of expressed CAZymes putatively targeting beta-glucans commonly found in diatom cell walls (88). It is not clear whether these systems were involved in diatom lysis or if they were activated following lysis; however, these observations supported our initial hypothesis that biopolymer degradation and the *Flavobacteriaceae* would be prevalent during algal demise (14, 17, 18).

During algal demise, the diversity of other putative biopolymer degraders present initially during growth was reduced, and only one other *Flavobacteriaceae* (*Muricauda* sp.) remained. We hypothesize that *Kordia* sp. removed competing bacteria, either by being optimized to demise conditions and/or by antibacterial modes (e.g., LodA and T6SS). However, several distinct genera of the *Rhodobacteraceae* co-occurred with *Kordia*, some assigned to the Marine Roseobacter Clade (e.g., *Roseovarius* and *Phaeobacter* [69, 70]). Significant metabolic versatility within the *Rhodobacteraceae* may confer adaptation to the widely ranging conditions along algal blooms (14, 18, 19). Nevertheless, the *Rhodobacteraceae* commonly specialize in the uptake of algal-released compounds during growth and development (20, 22). Specifically, *Rhodobacteraceae* taxa are enriched in transporters for dicarboxylic acids, sugars, dimethylsulfoniopropionate, and polyamines (71, 89, 90). Our analysis of polysaccharide CAZymes did not indicate any unique divergence from typical *Rhodobacteraceae* (71, 91, 92) to access carbon directly from biopolymers enriched during algal demise.

The appearance of distinct *Rhodobacteraceae* co-occurring with *Kordia* conflicted with our initial hypothesis that they would be more diverse during algal growth. Instead, our results collectively indicate that the co-occurrence of distinct *Rhodobacteraceae* with *Kordia* sp. during algal demise may be a consequence of cross-feeding. Cross-feeding is a ubiquitous phenomenon that structures microbial communities through cooperation (93), and previous studies have speculated that cross-feeding between *Rhodobacteraceae* and *Flavobacteriaceae* led to transparent exopolymer particle degradation within a diatom bloom (94). When biopolymers are abundant, community assembly generally follows “primary degraders” that initially colonize these resources (95, 96). Extracellular biopolymer hydrolysis releases oligo- and monomers into the environment, which are then consumed by “exploiters” (direct uptake) and/or “scavengers” (uptake of exuded metabolites [95, 97–99]). Indeed, based on proteomics transporter expression, *Rhodobacteraceae* members were actively transporting small molecules such as sugars and amino acids during algal pond demise, which may be a result of the degradation of biopolymers by *Kordia*. Our laboratory experiment supported this idea by demonstrating that the pre-processing of *P. tricornutum* lysate by *Kordia* (a primary degrader) allowed the growth of a representative Marine Roseobacter Clade strain (*Sulfitobacter* sp. N5S) under conditions analogous to algal demise. It is not known whether *Sulfitobacter* benefited from direct breakdown products, other exuded or released metabolites, or antimicrobial compound reduction. Yet, these results demonstrate how interspecies (inter-family in this case) interactions, driven by *Kordia*, can structure the microbiome. Previously, the addition of *Kordia algicida* to a natural phytoplankton community removed dominant species, released algal nutrients via lysis, and was correlated with increased dissolved organic carbon concentrations, possibly due to biopolymer degradation (100). It is possible that *Rhodobacteraceae* populations were exploiting diatom-stored nutrients released via lysis in the ARW ponds; however, our experimental system suggests that *Sulfitobacter* required *Kordia* conditioning to access DOM. A follow-up experiment might involve testing this hypothesis under mixed culture conditions (99) to investigate ecological interactions between *Kordia* and *Rhodobacteraceae*. Furthermore, the structuring of microbiota across the algal life cycle in general likely reflects different cross-feeding networks between microbes that exchange organic molecules and is not necessarily restricted to algal demise conditions.

Although many *Rhodobacteraceae* taxa emerged during algal demise, other *Rhodobacteraceae* were also present during the *P. tricornutum* growth stage, alluding to this group’s generalist lifestyle. We probed the genomic potential across 32 *Rhodobacteraceae* MAGs that were present in the *P. tricornutum* ponds to gain insight into potential metabolic strategies of this diverse and abundant community. Oxidation of the trace gas carbon monoxide (CO) can generate supplementary energy or carbon for persistence under “survival” conditions when other sources are limited (101, 102). For Roseobacter Clade members, the CO oxidation pathway is common and hypothesized to be used primarily for energy due to a lack of carbon fixation pathways (71). Here, we propose that CO may have been a viable source of energy, and potentially carbon, for the *Rhodobacteraceae* populations in the ponds. Indeed, a previous proteomic assessment done by our research group on *Rhodophyticola* sp. PT6CLA (*Rhodobacteraceae*) co-cultured with *P. tricornutum* in the laboratory revealed that CoxMSL was a highly expressed protein cluster (11), suggesting this may be a common biogeochemical transformation occurring in algal microbiomes. This suggests that CO oxidation may contribute significantly to carbon flow in algal ponds and could be a mechanism to reduce carbon emissions into the atmosphere, if the CO oxidation results in CO_2_ that is then fixed by microalgae. In addition, aromatic compound degradation pathways were also widespread among the *Rhodobacteraceae* MAGs, specifically for the degradation of coumaric acid to 4-hydroxybenzoate (4HBA) and 4HBA to protocatechuate, aromatics previously found to be exuded in abundance by *P. tricornutum* (16). Overall, these observations collectively emphasize the role of flexible carbon and energy metabolisms in structuring the microbiome within algal ponds and provide insight into potential survival mechanisms used by the *Rhodobacteraceae* when under competition for resources.

### Conclusions

Our characterization of a diatom population crash in outdoor ponds suggests *Kordia* sp. is both an environmentally and industrially relevant organism that can structure both algal and bacterial communities. Our analysis provides insight into potential virulence pathways employed by *Kordia* that could have led to *P. tricornutum* demise and/or the removal of bacterial competitors. Although we did not definitively demonstrate that *Kordia* activity directly led to the pond crash, the metagenomic and metaproteomic data highlight potential markers indicative of ponds that have a likelihood of crashing. This is not only informative for biofuel pond monitoring but also to better understand the state of the organic matter pool of crashed systems following the activity of microbes like *Kordia*. It is also relevant to the marine biological carbon pump that sequesters carbon through the sinking of dead phytoplankton, as *Kordia* and organisms with similar ecological roles can lead to algal lysis and control the microbial community that degrades algal lysates. Lastly, we highlight features of the globally relevant *Rhodobacteraceae* population, suggesting they are well adapted to respond to rapid changes in algal growth and sustain abundances using alternative carbon and energy sources.

## Data Availability

DNA/amplicon sequencing data are available through the JGI’s Integrated Microbial Genome database under accession codes in Table S1. MAG assemblies are available via Zenodo (https://doi.org/10.5281/zenodo.11414289). Bacterial strains are available upon request. Raw proteomics data are available on MassIVE (accession no. MSV000094933; https://massive.ucsd.edu/ProteoSAFe/dataset.jsp?accession=MSV000094933).

## SUPPLEMENTAL MATERIAL

Supplemental Material msystems.00375-25-s0001.docx Supplemental methods and figures.

Supplemental Tables msystems.00375-25-s0002.xlsx Tables S1-S9.

ASM does not own the copyrights to Supplemental Material that may be linked to, or accessed through, an article. The authors have granted ASM a non-exclusive, world-wide license to publish the Supplemental Material files. Please contact the corresponding author directly for reuse.
